# Canine parvovirus type 2 (CPV-2) and bacterial coinfections in dogs: phenotypic and genotypic resistance profiles in northern Kazakhstan

**DOI:** 10.3389/fvets.2025.1736817

**Published:** 2026-01-14

**Authors:** Yuliya Aleshina, Aigul Zhabykpayeva, Zulkyya Abilova, Yertay Yerzhanov, Andrey Nechaev, Daniyar Madiyev, Zhanaidar Bermukhametov, Raushan Rychshanova

**Affiliations:** 1 The Laboratory of Clinical Diagnostic, Microbiological Research and Safety of Materials of Biological Origin, Research Institute of Applied Biotechnology, Kostanay Regional University named after Akhmet Baitursynuly, Kostanay, Kazakhstan; 2 Department of Veterinary Medicine, Kostanay Regional University named after Akhmet Baitursynuly, Kostanay, Kazakhstan; 3 Department of General, Private and Operative Surgery, Saint Petersburg State University of Veterinary Medicine, St. Petersburg, Russia

**Keywords:** antimicrobial resistance, Central Asia, coinfection, CPV-2, cross-border surveillance, dogs, one health, resistance genes

## Abstract

**Introduction:**

Canine parvovirus type 2 (CPV-2) poses a serious viral threat to dogs. Despite the potential contribution of companion animals to antimicrobial resistance, data on CPV and associated bacterial coinfections remain limited. This study aimed to characterize the CPV-2 epizootic situation in Kostanay (Kazakhstan), assess bacterial coinfections and AMR profiles, including molecular markers, and evaluate their relevance to One Health.

**Methods:**

Epizootiological monitoring revealed a CPV-2 positivity rate of 19.4% (*n* = 549). For microbiological and PCR studies, two groups were established: CPV-2^+^ (*n* = 198) and clinically healthy (*n* = 200) dogs. *E. coli*, *Klebsiella* spp., and *S. aureus* were identified by culture/MALDI-TOF; AMR was assessed by disc diffusion (CLSI). Resistance genes were detected by PCR: for *Enterobacteriaceae*, blaTEM, OXA, tetA/tetB, StrA/StrB, aadB, aphA1, qnr/qepA, and sul1/sul3; for *S. aureus*, blaZ, ermB/ermC, tetK/tetM, and mecA.

**Results:**

A total of 131 isolates were obtained (CPV-2^+^: 72; healthy: 59), predominantly *E. coli* (*n* = 65) and *S. aureus* (*n* = 53). CPV-2^+^ dogs tended to carry more gram-negative bacteria. *Enterobacteriaceae* were highly resistant to tetracyclines (58.4%) and fluoroquinolones (51.2%), and sporadic carbapenem resistance was detected in *Klebsiella* (7%). *β*-Lactamase determinants (blaTEM/OXA) and aminoglycoside resistance genes were more frequently detected in CPV-2^+^ isolates, whereas blaZ predominated in *S. aureus*.

**Conclusion:**

CPV-2 infection is associated with a significant bacterial burden and pronounced AMR, supporting the need for improved surveillance and empirical therapy optimization. These results fill a geographical data gap in Central Asia and align with AMR trends reported in Europe and Asia, underscoring the cross-border circulation of CPV-2 and resistant bacteria and the need for a One Health approach.

## 1 Introduction

Canine parvovirus type 2 (CPV 2) was first identified in the late 1970s and quickly became one of the most important causative agents of infectious diseases in dogs (1, 2). Despite the widespread use of effective vaccines, 50 years after its identification, parvovirus remains a dangerous pathogen and continues to pose a serious threat to young dogs (2, 3). The virus causes severe forms of enteritis with haemorrhagic diarrhoea and myocarditis in puppies and is highly contagious and lethal, with a mortality rate of up to 91% in unvaccinated animals (2). CPV 2 has high mutational activity and has several antigenic variants (2a, 2b, and 2c), thanks to which it has retained its epidemiological significance despite the widespread introduction of vaccines (1). The virus affects the proliferating tissues of the intestine, lymphoid system and bone marrow, causing vomiting, haemorrhagic diarrhoea, depression, fever and dehydration, especially in puppies aged 6 weeks to 6 months, regardless of breed (4–6).

This disease is accompanied by pronounced immunosuppression, which contributes to the development of secondary bacterial infections, significantly complicating the clinical course of the disease and worsening the prognosis for recovery (7). Among the microorganisms associated with complications of viral infections in dogs, opportunistic bacteria such as *Escherichia coli*, *Klebsiella pneumoniae*, *Staphylococcus aureus*, *Proteus* spp., *Enterobacter* spp., and *Streptococcus* spp. are notable (8, 9). These bacteria usually have varying degrees of virulence and can significantly increase the severity of pathological changes, contributing to the development of sepsis and multiple organ failure and increasing mortality among infected dogs (10, 11). It has been established that coinfections significantly worsen the clinical course of major viral diseases and require intensive antibiotic therapy (12).

However, one of the most difficult problems in treating viral diseases complicated by bacterial infections in dogs is the resistance of opportunistic bacteria to widely used antibacterial drugs (13). Antibiotic resistance of microorganisms is a serious problem in modern veterinary medicine, substantially limiting therapeutic options and forcing clinicians to use more aggressive and expensive antibiotics (14). To date, a number of studies have demonstrated an increase in the resistance of bacteria isolated from dogs to antibiotics of the tetracycline, aminoglycoside, fluoroquinolone and cephalosporin classes (15). These observations underscore the need for regular monitoring of the antibiotic sensitivity of infectious agents in animals and the optimization of treatment regimens on the basis of the data obtained (16, 17).

In recent years, particular attention has been given to studying the antibiotic resistance genes carried by opportunistic bacteria that complicate the course of viral infections in dogs. It has been established that microorganisms such as *Escherichia coli*, *Klebsiella pneumoniae*, and *Staphylococcus aureus* may carry genes encoding resistance to beta-lactam antibiotics (blaTEM, blaSHV, and blaCTX-M), tetracyclines (tetA and tetB), aminoglycosides (aac(3)-IIa and aph(3′)-Ia) and fluoroquinolones (qnrA, qnrB, and qnrS) (18, 19). The presence of such genes not only limits therapeutic options but also contributes to the rapid spread of resistant strains in animal populations. In Kazakhstan, data on the prevalence of resistance genes in bacteria associated with viral infections in dogs are extremely limited, highlighting the need for further research in this area. The inclusion of molecular analysis in the comprehensive study of infectious diseases will allow a more accurate assessment of the risks of ineffective therapy and the development of strategies for the rational use of antibiotics in veterinary practice.

Current data indicate that domestic animals, especially dogs and cats, are important reservoirs of bacteria with multiple antimicrobial resistance (AMR) that can be transmitted between animals and humans. A recent study covering hundreds of clinical isolates from dogs and cats revealed that up to 75% of the isolates were resistant to at least one antibiotic and that a significant proportion were exhibited multidrug resistance (MDR), including resistance to penicillins and fluoroquinolones (20). In addition, a large retrospective study based on >2,500 urine samples from dogs and bacterial susceptibility testing revealed that *Escherichia coli* and other gram-negative pathogens are often resistant to first-line therapies, increasing the risk of treatment failure and complications (21).

Given that dogs infected with canine parvovirus type 2 (CPV-2) often develop immunosuppression and impaired intestinal barrier function, the risk of the colonization and translocation of resistant microorganisms increases. Therefore, a comprehensive study—considering the phenotypic profile of antibiotic sensitivity and molecular markers of resistance—is justified and necessary to assess the real threat of resistant infections, select the correct therapy, and prevent the spread of AMR in keeping with the One Health concept.

Thus, in this study, an epizootiological analysis of the CPV-2 incidence in Kostanay during the period 2020–2024 is combined with a study of the accompanying microflora and antimicrobial resistance genes in dogs with parvovirus enteritis and clinically healthy animals. This comprehensive approach allows us to assess the potential of opportunistic bacteria as markers of disease severity and to identify local resistance patterns that are crucial for optimizing prevention and treatment strategies for parvovirus enteritis.

## 2 Materials and methods

### 2.1 Diagnostics

The study was conducted at veterinary clinics in the city of Kostanay, as well as at the Laboratory of Clinical, Diagnostic and Microbiological Research and the Laboratory of Molecular and Genetic Analysis of the Research Institute of Applied Biotechnology at the Akhmet Baitursynov Kostanay Regional University, Kostanay, Republic of Kazakhstan.

To assess the epizootic spread of parvovirus infection among dogs between January 2020 and December 2024, 549 dogs of various breeds, aged between 1 month and 1 year, with clinical symptoms of gastrointestinal disease, were examined. A total of 198 dogs were diagnosed with parvovirus enteritis.

To diagnose parvovirus enteritis, the following evaluation was performed: collection of the medical history of the animal (age, sex, dietary habits, and vaccination and deworming status) and the disease (nature of the disease, whether the animal had been ill before and whether it had been treated with antibacterial drugs); physical examination for the presence of pathognomonic symptoms of the disease (apathy, refusal to eat, vomiting, diarrhoea, hyperthermia, and dehydration); and laboratory tests (morphological and biochemical blood tests). In addition, an ultrasound examination was performed. The final diagnosis of dogs suspected of having parvovirus enteritis was established on the basis of a comprehensive diagnostic approach that included immunochromatographic analysis to detect CPV-2 antigens (CPV-2/2a/2b/2c) in faecal samples, as well as confirmatory polymerase chain reaction (PCR) with viral DNA detection. The use of two complementary techniques improved the diagnostic accuracy and minimized the risk of false negative results in the early stages of infection.

CPV-2 was diagnosed using real-time polymerase chain reaction (real-time PCR).

Before extraction, the faecal samples were thoroughly homogenized in saline solution to obtain a 10% suspension. After a brief settling period, the supernatant was used for DNA extraction. When a rectal swab was used, the swab was transferred to 500 μL of a physiological solution and centrifuged to precipitate the particles.

For molecular detection, a conserved region of the VP2 structural gene characteristic of canine parvovirus type 2 (CPV-2) was used.

### 2.2 PCR methodology

The following primers, selected on the basis of published diagnostic protocols (22, 23), were used for CPV-2 DNA amplification:

- Forward (F): 5′-CAGGAAGATATCCAGAAGGA-3′.
- Reverse (R): 5′-GGTGCTAGTTGATATGTAATAAACA-3′.

Target region: VP2 gene fragment; amplicon length: 583 bp.

A 25 μL reaction mixture was prepared according to the test system manufacturer’s instructions and included 5 μL of isolated DNA.

Amplification method:

- initial denaturation: 95 °C for 3 min;
- denaturation: 40 cycles at 95 °C for 10 s + annealing and elongation at 60 °C for 30 s (with fluorescence detection in the FAM channel).

Positive, negative and internal controls were used in each reaction to confirm the validity of the results. The analytical sensitivity of the test system was at least 10^3^ genome equivalents/ml.

### 2.3 Blood tests

Haematological analyses were performed using an Exigo 17 veterinary haematology analyser (Spånga, Sweden), and 18 parameters were measured. Biochemical analyses were conducted in a BioChem FC-120 automated biochemical analyser (High Technology Inc., North Attleborough, MA, USA), which assessed 18 parameters, including potassium, phosphorus, sodium, urea, and creatinine. The reference ranges automatically established by each analyser were used as the standard values for blood parameters.

### 2.4 Microbiological studies

To assess the impact of parvovirus infection on the frequency of carriage and shedding of opportunistic microorganisms, two comparable groups of dogs were included in the study:

- Group 1: 198 dogs under 1 year of age with confirmed parvovirus enteritis (CPV-2).
- Group 2: clinically healthy dogs (*n* = 200), comparable in age/weight/breed to the dogs in Group 1.

Exclusion criteria: antibacterial/antifungal therapy within 30 days; severe stomatitis/rhinitis/diarrhoea (for enrolment in Group 2); intake of probiotics ≥10^9^ CFU/day in the past 7 days; and owner refusal.

Before admission to the hospital, clinical swabs were collected from all the dogs immediately upon initial entry, before placement in the reception area or treatment room. Samples were collected under aseptic conditions using standard sterile swabs. Biomaterial (from the oral cavity/gums, oropharynx, nasopharynx, and rectum) was collected for the isolation and identification of opportunistic microorganisms using a sterile swab, which was pressed firmly against the mucous membrane and rotated evenly for 5–10 s. The time from sampling to culture was ≤4 h. Negative control samples and standardized culture methods were used to control for contamination. If the phenotypic characteristics of bacteria isolated from clinical samples matched those of isolates from the clinic environment, we performed molecular typing to confirm their genetic relationship.

Pure cultures of microorganisms were isolated and grown using universal chromogenic and differential diagnostic culture media. Species identification of the isolates was performed using a MALDI Biotyper sirius RUO microbiological analyser.

The initial growth of microorganisms was carried out by seeding the material in meat peptone broth (MPB) prior to incubation at 36–37 °C for 18–24 h. Afterwards, the material was transferred to universal and chromogenic differential diagnostic culture media (including CHROMagar™ for enterobacteria and staphylococci) and incubated at 36–37 °C for 18–24 h. After the appearance of characteristic colonies, macroscopic evaluation of growth (colour, shape, size, haemolysis, and surface characteristics) and microscopy of Gram-stained smears were performed for preliminary differentiation of gram-positive and gram-negative bacteria.

Pure cultures were obtained by repeated subculturing of isolated typical colonies and incubation under the same conditions until single-strain growth was accomplished. Species identification of *Escherichia coli*, *Klebsiella* spp. and *Staphylococcus aureus* was performed using the MALDI Biotyper sirius RUO microbiological analyser (Bruker, Germany) in accordance with the manufacturer’s instructions.

### 2.5 Antibiotic sensitivity testing

The antimicrobial sensitivity of isolated bacterial strains of *E. coli*, *Klebsiella* spp., and *S. aureus* was assessed using the disc diffusion method (Kirby–Bauer) on Mueller–Hinton agar in accordance with the recommendations of the Clinical and Laboratory Standards Institute (CLSI) (24). A standardized bacterial suspension was prepared to a density of 0.5 on the McFarland scale, after which it was evenly applied to the surface of agar plates with a sterile swab. The plates were incubated at 35–37 °C for 18–24 h; then, the sensitivity model was evaluated by measuring the diameter of the inhibition zone, and the isolates were considered resistant, intermediate, or susceptible according to the CLSI ranges (24). The *E. coli* ATCC 25922 and *S. aureus* ATCC 25923 reference strains were used to control the quality of the media used and the correctness of the test setup.

For testing of microorganisms of the Enterobacteriaceae family, a panel of 17 antibiotics was used: amoxicillin, 25 μg; ampicillin, 10 μg; cefoperazone, 75 μg; cefoxitin, 30 μg; cefpodoxime, 10 μg; meropenem, 10 μg; streptomycin, 10 μg; kanamycin, 30 μg; gentamicin, 10 μg; tetracycline, 15 μg; doxycycline, 30 μg; enrofloxacin, 5 μg; ciprofloxacin, 5 μg; norfloxacin, 10 μg; ofloxacin, 5 μg; gemifloxacin, 5 μg; and sulfamethoxazole/trimethoprim, 23.75 μg/1.25 μg. For testing of *S. aureus*, a panel of 16 antibiotics was used: amoxicillin, 25 μg; ampicillin, 10 μg; penicillin, 10 μg; cefoperazone, 75 μg; cefoxitin, 30 μg; streptomycin, 10 μg; kanamycin, 30 μg; neomycin, 30 μg; gentamicin, 10 μg; tetracycline, 30 μg; doxycycline, 30 μg; erythromycin, 15mcg; tylosin, 15mcg; sulfamethoxazole/trimethoprim, 23.75 mcg/1.25 mcg; ciprofloxacin, 5 mcg; and norfloxacin, 10 mcg.

To prevent contamination and cross-contamination of samples, the principle of unidirectional laboratory flow was strictly observed: the processes of sample reception and registration, seeding, incubation and identification were carried out in separate areas. All manipulations were carried out under aseptic conditions using disposable sterile consumables (swabs, loops, and filter tips), which were replaced for each new sample. The work surfaces were treated with disinfectant solutions before and after work and were also subjected to ultraviolet irradiation in accordance with laboratory regulations.

### 2.6 Identification of antibiotic resistance genes by PCR

Genomic DNA from phenotypically identified microbial colonies was extracted by the boiling method using PureLink Genomic DNA Kits (Thermo Fisher Scientific, USA) according to the manufacturer’s instructions and was then stored at −20 °C until further analysis. For genotypic analysis of the strains, genes associated with resistance to *β*-lactam antibiotics (*blaTEM, OXA, blaZ, mecA*), aminoglycosides (*strA, strB, aadB, aphA1, aac(6′)-aph(2″),* and *aph(3′)*), tetracyclines (*tetA, tetB, tetK,* and *tetM*), sulfonamides (*sul1* and *sul3*), trimethoprim (*dfrG* and *dfrK*), fluoroquinolones (*qepA* and *qnr*), and macrolides (*ermC* and *ermB*) were targeted. Primers were selected considering the antibiotic and antimicrobial classes most commonly used in veterinary practice (Table 1). The synthesis of primers and fluorescently labelled probes was performed at the National Center for Biotechnology (Astana, Kazakhstan; Z05K8A3).

**Table 1:** Primers used in the study.

Bacterium	Primer sequence (5′–3′)	Target gene	Amplicon size (bp)	References
*E. coli*, *Klebsiella* spp.	ATCAGTTGGGTGCACGAGTG	*BlaTEM*	608	Chuanchuen et al. (25)
	ACGCTCACCGGCTCCAGA			
	ATGAAAAACACAATACATATCAAC	*OXA*	755	Edelstein et al. (26)
	AAAGGACATTCACGCCTGTG			
	CCAATCGCAGATAGAAGGC	*StrA*	546	Scholz et al. (27)
	CTTGGTGATAACGGCAATTC			
	GGATCGTAGAACATATTGGC	*StrB*	509	Scholz et al. (27)
	ATCGTCAAGGGATTGAAACC			
	CTAGCTGCGGCAGATGAGC	*aadB*	300	Asadollahi et al. (28)
	CTCAGCCGCCTCTGGGC			
	AAACGTCTTGCTCGAGGC	*aphA1*	500	Guerra et al. (29)
	CAAACCGTTATTCATTCGTGA			
	GCTACATCCTGCTTGCCT	*tetA*	210	Asai et al. (30)
	CATAGATCGCCGTGAAGA			
	CATTAATAGGCGCATCGCTG	*tetB*	930	Rather et al. (31)
	TGAAGGTCATCGATAGCAGG			
	CTTCGATGAGAGCCGGCGGC	*SUL1*	436	Guerra et al. (29)
	GCAAGGCGGAAACCCCGCC			
	GAGCAAGATTTTTGGAATCG	*SUL3*	500	Perreten et al. (32)
	CATCTGCAGCTAACCTAGGGCTTTGGA			
	GCAGGTCCAGCAGCGGGTAG	*qepA*	218	Liu et al. (33)
	CTTCCTGCCCGAGTATCGTG			
	ATTTCTCACGCCAGGATTTG	*qnr*	516	Robicsek et al. (34)
	GATCGGCAAAGGTTAGGTCA			
*S. aureus*	CAGTTCACATGCCAAAGAG	*blaZ*	772	Schnellmann et al. (35)
	TACACTCTTGGCGGTTTC			
	GGGATCATAGCGTCATTATTC	*mecA*	527	Couto et al. (36)
	AACGATTGTGACACGATAGCC			
	CAGAGCCTTGGGAAGATGAA	*aac(6)-aph2*	348	Couto et al. (36)
	CCTCGTGTAATTCATGTTCTGG			
	CCGCTGCGTAAAAGATA	*aph(3)*	609	Perreten et al. (37)
	GTCATACCACTTGTCCGC			
	TTAGGTGAAGGGTTAGGTCC	*tetK*	718	Strommenger et al. (38)
	GCAAACTCATTCCAGAAGC			
	GTTAAATAGTGTTCTTGGAG	*tetM*	686	Strommenger et al. (38)
	CTAAGATATGGCTCTAACAA			
	TTTCTTTGATTGCTGCGATG	*dfrG*	1,230	Couto et al. (36)
	AACGCACCCGTTAACTCAAT			
	GCTGCGATGGATAAGAACAG	*dfrK*	214	Couto et al. (36)
	GGACGATTTCACAACCATTAAAGC			
	ATCTTTGAAATCGGCTCAGG	*ermC*	292	Couto et al. (36)
	CAAACCCGTATTCCACGAT			
	GAAAAGGTACTCAACCAAATA	*ermB*	*639*	Sutcliffe et al. (39)
	AGTAACGGTACTTAAATTGTTTAC			

### 2.7 Identification of *E. coli* serogroups and identification of Enterobacteriaceae virulence genes

To identify toxin-producing *E. coli*, 24-h cultures of the microorganisms were streaked onto the chromogenic media CHROMagar™ STEC and CHROMagar™ O157 (CHROMagar, France) for the detection of Shiga toxin-producing *E. coli* (STEC) and for the qualitative identification of *E. coli* serotype O157: H7. The plates were incubated for 18–24 h at 37 °C, after which the colonies were examined and identified.

### 2.8 Detection of *Staphylococcus aureus* enterotoxins (a to E)

For the detection of *Staphylococcus aureus* enterotoxins in bacterial cultures, a commercial enzyme-linked immunosorbent assay (ELISA) kit for the combined detection of enterotoxins A–E (RIDASCREEN^®^ SET Total, R-Biopharm AG, Germany) was used.

*S. aureus* isolates obtained on selective media were identified according to standard microbiological criteria and were then precultured in brain heart infusion (BHI) broth to optimize enterotoxin production. A cell suspension was subsequently prepared following the manufacturer’s instructions, and culture supernatants were used for analysis.

Samples and control solutions were added to wells precoated with specific antibodies against enterotoxins A–E. Incubation was performed according to the kit protocol. After the samples were washed, the antibody conjugate was added, after which the chromogenic substrate was added. The reaction was stopped by the addition of stop solution, and the optical density (OD) was measured at 450 nm using an ELISA reader. A positive result was defined according to the manufacturer’s criteria: the sample OD value exceeded the threshold calculated from the negative control.

Using this ELISA kit, enterotoxins SEA, SEB, SEC, SED, and SEE were simultaneously detected in culture supernatants of *S. aureus* isolates obtained from dogs in both study groups.

### 2.9 Statistical processing of results

The study power was calculated to compare two independent groups with an expected difference in the frequency of bacterial coinfection of at least 15% at a significance level of *α* = 0.05 and a statistical power of 80%. On the basis of the calculation, the minimum required sample size per group was ≥180 animals.

Descriptive statistics were used for data processing: quantitative indicators (haematological and biochemical parameters) are presented as the means ± standard deviations for normally distributed data or the medians with interquartile ranges for nonnormally distributed data. The frequency indicators (frequency of microorganism isolation and presence of resistance genes) are presented as absolute and percentage values.

Pearson’s *χ*^2^ test was used to compare the frequency of microorganism isolation between the dogs with CPV-2 and the clinically healthy animals, and Fisher’s exact test was used for small sample sizes. A *p* value of < 0.05 was considered to indicate statistical significance.

To assess the correlations between the presence of resistance genes and phenotypic resistance, a 2 × 2 contingency table was constructed for each gene and the corresponding class of antimicrobial drugs. The sensitivity phenotype was interpreted according to the categories S/I/R (sensitive/intermediate/resistant); in the statistical analysis, only isolates in the R category were considered “resistant”, while the isolates in the S and I categories were combined into the “nonresistant” group. The presence or absence of resistance genes was determined by PCR. For each gene–phenotype pair, the proportion of correspondence (%), odds ratio (OR) with 95% confidence interval, and Fisher’s exact test *p* values were calculated to assess the statistical significance of the differences. For tables with zero values in the cells, the Haldane–Anskombs correction (adding 0.5) was applied. The criterion for statistical significance was considered to be *p* < 0.05.

All calculations were performed using Microsoft Excel 2019 and Statistica v.13.

### 2.10 Ethical approval

This study was conducted in accordance with the principles of ethical research involving animals, as outlined in the National Institutes of Health Guide for the Care and Use of Laboratory Animals. All animal procedures were approved by the Institutional Animal Care and Use Committee (IACUC). Blood samples were collected from dogs with the informed consent of their owners and in compliance with ethical guidelines for the humane treatment of animals. The data obtained in this study will be used solely for scientific purposes and will be presented with respect for the confidentiality and privacy of both the dogs and their owners.

## 3 Results

### 3.1 Epizootiological monitoring

Between January 2020 and December 2024, veterinary clinics in the city of Kostanay treated 2,831 dogs. Of these dogs, 549 presented with symptoms of gastrointestinal tract diseases at registration. Laboratory diagnostics (PCR and immunochromatographic testing) confirmed parvovirus enteritis in 7% (*n* = 198) of the total number of registered dogs. According to clinical records, 80% of these puppies had not received any vaccinations, and 20% had received only one dose of the vaccine, after which they subsequently developed clinical signs of parvovirus enteritis. There were no fully vaccinated animals in the study population. Thus, the group was predominantly unvaccinated, which minimized the variability associated with vaccine-induced immunity. Additionally, none of the dogs had received antibiotics prior to admission to the clinic.

The annual incidence rates are presented in Table 2.

**Table 2:** Temporal dynamics of parvoviral enteritis incidence in dogs in Kostanay, 2020–2024.

Year	Number of cases	Absolute		Retention rate (%)	Growth rate (%)	Rate of change (%)	
		Increase	Decrease			Increase	Decrease
2020	103	None		100.0	NO	NO	
2021	128	+ 25.0		124.3	124.3	+ 24.3	
2022	115		−13.0	111.7	89.8		−10.2
2023	109		−6.0	105.8	94.8		−5.2
2024	94		−15.0	91.3	86.2		−13.8

The absolute increase or decrease was calculated relative to the previous year.

The highest number of cases was recorded in 2021 (128 cases). After 2021, there was a steady decreasing trend in incidence. From 2020 to 2024, the total number of cases decreased by 8.7% (visibility index 91.3%).

As part of the epizootiological monitoring, the outcome of the disease in puppies with confirmed CPV-2 was assessed, with an outcome of fatality recorded for 38 of the 198 dogs (19.2%).

### 3.2 Microbiological studies

Two groups of dogs were established for microbiota analysis: Group 1 (*n* = 198) consisted of dogs under 1 year of age with PCR-confirmed parvovirus enteritis (CPV-2^+^), and Group 2 (*n* = 200) consisted of clinically healthy dogs (control group). The main risk factor in Group 1 was a lack of vaccination. The species composition and frequency of bacterial isolation are presented in Table 3.

**Table 3:** Bacterial species composition and frequency of isolation in dogs with parvovirus enteritis and healthy dogs.

Microorganisms	CPV-2^+^ (*n* = 198)	Healthy dogs (*n* = 200)	Total isolates	Тест	*p*-value
*E. coli*	17,7% (35/198)	15,0% (30/200)	65	*χ*^2^ = 0.52	0.47
*S. aureus*	14,6% (29/198)	12,0% (24/200)	13	*χ*^2^ = 0.60	0.44
*Klebsiella* spp.	4,0% (8/198)	2,5% (5/200)	53	Fisher	0.41
Total	35,4% (72/198)	29,5% (59/200)	131	*χ*^2^ = 1.32	0.25

A greater number of bacterial isolates (*n* = 72) were obtained from dogs with parvovirus enteritis than from healthy dogs (*n* = 59), indicating increased bacterial contamination associated with viral infection. The same bacterial species were predominant in both groups: *E. coli*, *S. aureus*, and *Klebsiella* spp.

Comparison of the frequency of isolation between the groups revealed that gram-negative bacteria were isolated slightly more often in dogs infected with CPV-2 (*n* = 198) than in clinically healthy animals (35.4% vs. 29.5%; *χ*^2^ = 1.32; *p* = 0.25; 95%). No statistically significant differences were detected in the analysis of individual genera: *E. coli* –17.7% vs. 15.0% (*χ*^2^ = 0.52; *p* = 0.47; 95% CI: −4.6–9.9 p.p.), *S. aureus*—14.6% vs. 12.0% (*χ*^2^ = 0.60; *p* = 0.44), and *Klebsiella* spp. –4.0% vs. 2.5% (*p* = 0.41, Fisher’s exact test). Thus, although higher absolute values of bacterial isolation frequency were observed in the CPV-2^+^ group, the differences were not statistically significant.

### 3.3 Clinical status of animals with parvovirus enteritis complicated by infections with opportunistic microorganisms

Most dogs with confirmed CPV-2 showed a characteristic clinical profile of acute viral enteritis. The frequencies of key symptoms are shown in Table 4.

**Table 4:** Frequency of clinical symptoms in dogs with CPV2 (*n* = 126).

Clinical sign	Frequency, %
Lethargy, apathy	100%
Mucohaemorrhagic diarrhoea	100%
Repeated vomiting	87%
Dehydration, hyperthermia	75%
Asthenia	78%
Decreased skin turgor	79%
Anorexia	58%
Pallor of visible mucous membranes	63%
Tachycardia	45%
Weak pulse	67%
Abdominal pain	50%
Intestinal atony	41%
Prolonged capillary refill, muffled heart sounds, paresis	Observed occasionally

The most common symptoms were pronounced lethargy, apathy, and mucohaemorrhagic diarrhoea, which were observed in all the animals. Repeated vomiting, signs of dehydration, and hyperthermia were observed in most patients, whereas anorexia, pale mucous membranes, tachycardia, and weak pulse were less common but also clinically significant. Less frequently, intestinal atony, abdominal wall tenderness, and isolated signs of systemic involvement, including prolonged capillary refill and muffled heart sounds, were recorded.

Comparative analysis of the clinical and laboratory data revealed that the severity of parvovirus enteritis varied significantly depending on the type of bacterial coinfection. The detailed differences in clinical signs and laboratory parameters are shown in Table 5.

**Table 5:** Clinical, haematological, and biochemical characteristics of CPV-2-induced enteritis depending on the isolated microorganism.

Indicator	CPV-2 + *Escherichia coli* (*n* = 35)	CPV-2 + *Klebsiella* spp. (*n* = 8)	CPV-2 + *S.aureus* (*n* = 29)
General course of the disease	Severe gastrointestinal tract damage	Most severe cases, septic course	Moderately severe cases, protracted recovery
Key clinical symptoms	Intense mucohemorrhagic diarrhoea; profuse vomiting; severe dehydration; hyperthermia; rapid exhaustion	Severe intoxication and depression; hyperthermia up to 40.5 °C; foul-smelling mucohemorrhagic diarrhoea; shortness of breath; pallor/cyanosis of the mucous membranes	Moderate fever; vomiting bile; weakness and anorexia; abdominal distension and tenderness; muffled heart sounds; pale mucous membranes

Haematology			
Haemoglobin, g/l	98 ± 12	88 ± 10	112 ± 11
Leukocytes, ×10^9^/L	3.1 ± 0.9	2.0 ± 0.6	4.2 ± 1.1
Neutrophils, ×10^9^/L	1.5 ± 0.7	0.9 ± 0.4	2.0 ± 0.8
Thrombocytes, ×10^9^/L	155 ± 38	108 ± 32	168 ± 42
Total protein, g/L	44 ± 5	39 ± 4	48 ± 6
Albumin, g/L	21 ± 3	18 ± 3	23 ± 4
ESR, mm/h	14 ± 5	18 ± 7	16 ± 6

Biochemistry			
Glucose, mmol/L	3.0 ± 0.6	2.8 ± 0.5	3.5 ± 0.7
Potassium, mmol/L	3.0 ± 0.4	2.9 ± 0.4	3.3 ± 0.3
Sodium, mmol/L	138 ± 4	136 ± 5	140 ± 4
ALT, U/L	82 ± 18	96 ± 22	78 ± 15
AST, U/L	74 ± 21	88 ± 24	66 ± 18
LDH, U/L	420 ± 90	505 ± 110	385 ± 80
Urea, mmol/L	10.2 ± 3.4	16.5 ± 4.2	8.8 ± 2.8
Creatinine, μmol/L	135 ± 30	185 ± 40	125 ± 28
Interpretation of condition	Intestinal barrier disruption, significant protein and electrolyte loss, toxic effects of *E. coli*	High risk of multiple organ failure and DIC syndrome (septic nature of complications)	Slow regeneration of the mucous membrane, prolonged recovery

The most pronounced disorders were observed in dogs with CPV-2 and *Klebsiella* spp. infection, which was accompanied by a septic course, profound haematological shifts, pronounced hypoproteinaemia, and increased renal and hepatic dysfunction, as indicated by the valuer of these markers. Animals with CPV-2 + *E. coli* infection had severe gastrointestinal tract damage, significant electrolyte and protein loss, and pronounced leukopaenia. Coinfection with *S. aureus* presented a more moderate clinical picture but was characterized by a protracted inflammatory process and a longer recovery period. The results of the combined clinical, haematological, and biochemical analysis emphasize that the type of bacterial agent significantly affects the severity of the disease and the prognosis.

### 3.4 Antibiotic resistance of opportunistic microorganisms

The antibiotic resistance of opportunistic microorganisms isolated from dogs with parvoviral enteritis (*n* = 198) and from clinically healthy animals (*n* = 200) was investigated. The results revealed a high level of multidrug resistance (MDR), particularly among isolates from diseased animals (Table 5).

The phenotypic resistance rates of *E. coli* and *Klebsiella* spp. isolated from dogs with parvovirus enteritis and clinically healthy animals to the main classes of antimicrobial drugs are shown in Table 6.

**Table 6:** Comparative antibiotic resistance of enterobacteria isolated from diseased and healthy dogs (%).

Antibiotic	Bacterium	CPV-2^+^, *R*/n* (%)	Healthy, *R*/n* (%)
Ampicillin	*E. coli*	7/35 (20.0%)	10/30 (33.3%)
	*Klebsiella*	8/8 (100%)	3/5 (60.0%)
Amoxicillin	*E. coli*	7/35 (20.0%)	8/30 (26.7%)
	*Klebsiella*	8/8 (100%)	4/5 (80.0%)
Cefoperazone	*E. coli*	5/35 (14.3%)	5/30 (16.7%)
	*Klebsiella*	5/8 (62.5%)	1/5 (20.0%)
Cefotaxime	*E. coli*	5/35 (14.3%)	3/30 (10.0%)
	*Klebsiella*	5/8 (62.5%)	3/5 (60.0%)
Cefpodoxime	*E. coli*	9/35 (25.7%)	5/30 (16.7%)
	*Klebsiella*	5/8 (62.5%)	3/5 (60.0%)
Meropenem	*E. coli*	0/35 (0%)	2/30 (6.7%)
	*Klebsiella*	3/8 (37.5%)	0/5 (0%)
Streptomycin	*E. coli*	0/35 (0%)	1/30 (3.3%)
	*Klebsiella*	2/8 (25.0%)	0/5 (0%)
Kanamycin	*E. coli*	1/35 (2.9%)	2/30 (6.7%)
	*Klebsiella*	3/8 (37.5%)	0/5 (0%)
Gentamicin	*E. coli*	2/35 (5.7%)	4/30 (13.3%)
	*Klebsiella*	4/8 (50.0%)	0/5 (0%)
**Tetracycline**	*E. coli*	**20/35 (57.1%)**	**7/30 (23.3%)**
	*Klebsiella*	5/8 (62.5%)	3/5 (60%)
Doxycycline	*E. coli*	11/35 (31.4%)	7/30 (23.3%)
	*Klebsiella*	5/8 (62.5%)	3/5 (60%)
Enrofloxacin	*E. coli*	5/35 (14.3%)	5/30 (16.7%)
	*Klebsiella*	3/8 (37.5%)	1/5 (20%)
Ciprofloxacin	*E. coli*	9/35 (25.7%)	4/30 (13.3%)
	*Klebsiella*	3/8 (37.5%)	1/5 (20%)
Ofloxacin	*E. coli*	12/35 (34.3%)	7/30 (23.3%)
	*Klebsiella*	5/8 (62.5%)	2/5 (40%)
Hemifloxacin	*E. coli*	3/35 (8.6%)	2/30 (6.7%)
	*Klebsiella*	2/8 (25%)	1/5 (20%)
TMP/SMX	*E. coli*	5/35 (14.3%)	6/30 (20%)
	*Klebsiella*	5/8 (62.5%)	3/5 (60%)

*R, number of resistant strains; *n*, total number of microorganisms of this species.

*E. coli* isolates obtained from dogs with parvovirus enteritis exhibited the highest rates of resistance to tetracycline (57.1%) and ofloxacin (34.3%), followed by ciprofloxacin (25.7%) and cefpodoxime (25.7%). The rates of resistance to ampicillin and amoxicillin were 20.0%, while a low rate of resistance to aminoglycosides was observed (<6%). None of these isolates were resistant to meropenem (0%).

*E. coli* isolates from clinically healthy dogs also showed resistance mainly to fluoroquinolones (norfloxacin—43.3%, ofloxacin—23.3%), with lower rates of resistance to tetracyclines (23.3%) and cephalosporins (up to 16.7%).

*Klebsiella* spp. were highly resistant to *β*-lactams in both groups, with rates of resistance to ampicillin and amoxicillin of 100% in dogs with parvovirus and 60–80% in healthy dogs. Significant resistance to fluoroquinolones (up to 62.5%) and tetracyclines (60–62.5%) was also detected. Resistance to meropenem was detected only in animals infected with CPV-2 (37.5%).

Analysis of phenotypic resistance revealed that the proportions of resistant isolates of *E. coli* and *Klebsiella* spp. did not significantly differ between the group of dogs with CPV-2 and the group of clinically healthy animals (*p* > 0.05 for all microorganism–antibiotic combinations). The lone exception was tetracycline for *E. coli* isolates, for which a significantly higher prevalence of resistance was recorded in dogs infected with CPV-2 (57.1% vs. 23.3%; OR = 4.28; 95% CI: 1.46–12.89; *p* = 0.011).

Data on *S. aureus* resistance are presented in Table 7.

**Table 7:** Comparative antibiotic resistance of *Staphylococcus aureus* isolated from diseased and healthy dogs (%).

Antibiotic	CPV-2^+^, *R*/n* (%)	Healthy, *R*/n* (%)
Ampicillin	9/29 (31.0%)	9/24 (37.5%)
Penicillin	7/29 (24.1%)	8/24 (33.3%)
Amoxicillin	2/29 (6.9%)	1/24 (4.2%)
Cefoperazone	0/29 (0%)	2/24 (8.3%)
Cefoxitin	1/29 (3.4%)	0/24 (0%)
Streptomycin	2/29 (6.9%)	2/24 (8.3%)
Kanamycin	1/29 (3.4%)	1/24 (4.2%)
Neomycin	1/29 (3.4%)	2/24 (8.3%)
Gentamicin	0/29 (0%)	0/24 (0%)
Tetracycline	14/29 (48.3%)	4/24 (16.7%)
Tylosin	7/29 (24.1%)	2/24 (8.3%)
Erythromycin	8/29 (27.6%)	3/24 (12.5%)
Doxycycline	4/29 (13.8%)	2/24 (8.3%)
Ciprofloxacin	3/29 (10.3%)	3/24 (12.5%)
Norfloxacin	1/29 (3.4%)	1/24 (4.2%)
TMP/SMX	2/29 (6.9%)	2/24 (8.3%)

*R, number of resistant strains; *n*, total number of microorganisms of this species.

The table shows the resistance rates of staphylococci isolated from dogs with parvovirus enteritis and clinically healthy animals to antimicrobial drugs.

Staphylococci isolates from the group of dogs with parvovirus enteritis exhibited the highest rates of resistance to tetracycline (48.3%), erythromycin (27.6%), penicillin (24.1%) and tylosin (24.1%). Moderate resistance to ampicillin and trimethoprim/sulfamethoxazole (6.9% each) was detected. A low rate of resistance to aminoglycosides was observed (<6.9%), and no resistance to gentamicin was detected.

In the group of healthy dogs, the highest resistance rates were associated with penicillin (33.3%) and ampicillin (37.5%). The rate of tetracycline resistance (16.7%) was significantly lower than that of isolates from dogs with parvovirus, similar to the resistance profile for macrolides (8.3–12.5%).

Thus, *S. aureus* isolates from both groups were characterized by *β*-lactam resistance, but macrolide- and tetracycline-resistant phenotypes were more common in CPV-2^+^ animals.

Statistical analysis revealed that for most antibiotics, the differences between the groups were nonsignificant (*p* > 0.05). However, the incidence of tetracycline-resistant strains was significantly higher in the dogs with parvovirus enteritis (48.3% (14/29) vs. 16.7% (4/24); OR = 4.67; 95% CI: 1.28–17.08; *p* = 0.021).

### 3.5 Identification of antibiotic resistance genes by PCR

Tables 8, 9 present the distribution of genes associated with the resistance of opportunistic microorganisms to various classes of antibiotics.

**Table 8:** Antibiotic resistance genes detected in *Enterobacteriaceae* isolates obtained from dogs with parvovirus enteritis and healthy dogs.

Antibiotic class	Gene	CPV-2^+^, *R*/n* (%)		Healthy, *R*/n* (%)		Total, *R*/*n* (%)
		*E. coli*	*Klebsiella*	*E. coli*	*Klebsiella*	
β-Lactams	*BlaTEM*	7/35 (20.0%)	4/8 (50.0%)	4/30 (13.3%)	2/5 (40.0%)	17/78 (21.8%)
	*OXA*	5/35 (14.3%)	2/8 (25.0%)	3/30 (10.0%)	2/5 (40.0%)	12/78 (15.4%)
Amino-glycosides	*StrA*	4/35 (11.4%)	0/8 (0.0%)	2/30 (6.7%)	2/5 (40.0%)	8/78 (10.3%)
	*StrB*	4/35 (11.4%)	1/8 (12.5%)	1/30 (3.3%)	2/5 (40.0%)	8/78 (10.3%)
	*aadB*	1/35 (2.9%)	2/8 (25.0%)	2/30 (6.7%)	0/5 (0.0%)	5/78 (6.4%)
	*aphA1*	2/35 (5.7%)	2/8 (25.0%)	1/30 (3.3%)	1/5 (20.0%)	6/78 (7.7%)
Tetracyclines	*tetA*	3/35 (8.6%)	1/8 (12.5%)	4/30 (13.3%)	1/5 (20.0%)	9/78 (11.5%)
	*tetB*	3/35 (8.6%)	2/8 (25.0%)	2/30 (6.7%)	2/5 (40.0%)	9/78 (11.5%)
Sulfonamides	*SUL1*	2/35 (5.7%)	1/8 (12.5%)	1/30 (3.3%)	1/5 (20.0%)	5/78 (6.4%)
	*SUL3*	1/35 (2.9%)	0/8 (0.0%)	3/30 (10.0%)	1/5 (20.0%)	5/78 (6.4%)
Fluoro-quinolones	*qepA*	1/35 (2.9%)	2/8 (25.0%)	1/30 (3.3%)	0/5 (0.0%)	4/78 (5.1%)
	*qnr*	1/35 (2.9%)	1/8 (12.5%)	2/30 (6.7%)	1/5 (20.0%)	5/78 (6.4%)

*R, number of resistant strains; *n*, total number of microorganisms of this species.

**Table 9:** Antibiotic resistance genes detected in *Staphylococcus aureus* isolates obtained from dogs with parvovirus enteritis and healthy dogs.

Antibiotic group	Gene	CPV-2^+^, *R*/n* (%)	Healthy, *R*/n* (%)	Total, *R*/*n* (%)
*β*-Lactams	*BlaZ*	7/29 (24.1%)	5/24 (20.8%)	12/53 (22.6%)
	*mecA*	0/29 (0.0%)	0/24 (0.0%)	0/53 (0.0%)
Macrolides	*ermC*	3/29 (10.3%)	1/24 (4.2%)	4/53 (7.5%)
	*ermB*	2/29 (6.9%)	2/24 (8.3%)	4/53 (7.5%)
Aminoglycosides	*aac(6)-aph2*	1/29 (3.4%)	2/24 (8.3%)	3/53 (5.7%)
	*aph(3)*	2/29 (6.9%)	2/24 (8.3%)	4/53 (7.5%)
Tetracyclines	*tetK*	5/29 (17.2%)	2/24 (8.3%)	7/53 (13.2%)
	*tetM*	3/29 (10.3%)	1/24 (4.2%)	4/53 (7.5%)
Sulfonamides	*dfrG*	0/29 (0.0%)	0/24 (0.0%)	0/53 (0.0%)
	*dfrK*	0/29 (0.0%)	0/24 (0.0%)	0/53 (0.0%)

*R, number of resistant strains; *n*, total number of microorganisms of this species.

As shown in Table 8, analysis of resistance determinants revealed that the greatest number of genes detected were associated with resistance to *β*-lactams and aminoglycosides. In *E. coli* isolates from dogs with parvoviral enteritis, the most frequently detected genes were *blaTEM* (20.0%), *OXA* (14.3%), *tetB* (11.4%), and the aminoglycoside resistance genes *StrA* (11.4%) and *StrB* (11.4%).

In *E. coli* isolates from clinically healthy dogs, the predominant genes were *blaTEM* (16.7%), *tetA* (13.3%), *tetB* (6.7%), *aadB* (6.7%), and *qnr* (6.7%).

In *Klebsiella* spp. (*n* = 13), the most prevalent genes included *blaTEM* (46.2%), *OXA* (30.8%), *tetB* (30.8%), *aphA1* (23.1%), *qepA* (15.4%), and *SUL1/SUL3* (15.4% each).

Overall, *β*-lactamase genes (*blaTEM* and *OXA*) were detected in 30 cases (38.5% of all the isolates), aminoglycoside resistance genes (*StrA*, *StrB*, *aadB*, and *aphA1*) were detected in 28 cases (35.9%), tetracycline resistance genes (*tetA* and *tetB*) were detected in 19 cases (24.4%), sulfonamide resistance genes (*SUL1* and *SUL3*) were detected in 10 cases (12.8%), and fluoroquinolone resistance genes (*qepA* and *qnr*) were detected in 9 cases (11.5%).

Molecular analysis of *S. aureus* isolates revealed that genes encoding resistance to β-lactam antibiotics were the most frequently detected. The *blaZ* gene was detected in 12 isolates (22.6%) and was more frequently detected in dogs with parvoviral enteritis (24.1%) than in clinically healthy dogs (20.8%). The *mecA* gene, associated with methicillin resistance (MRSA), was not detected in any of the samples (Table 9).

Among the macrolide resistance genes, *ermC* (7.5%) and *ermB* (7.5%) were detected, and their distributions were similar between the groups. Aminoglycoside resistance was mediated by the genes *aac(6′)-aph(2″)* (5.7%) and *aph(3′)* (7.5%).

Tetracycline resistance genes were detected in a considerable number of isolates: *tetK* in 7 isolates (13.2%) and *tetM* in 4 isolates (7.5%). The prevalence of these genes was greater in dogs with parvoviral enteritis (*tetK*: 17.2%; *tetM*: 10.3%) than in clinically healthy dogs (*tetK*: 8.3%; *tetM*: 4.2%).

No sulfonamide resistance genes (*dfrG* or *dfrK*) were detected in the *S. aureus* isolates examined.

The correlations between the presence of resistance genes and phenotypic resistance in bacterial isolates from dogs was analysed (Table 10).

**Table 10:** Correlation analysis between genotype and phenotype.

Gene	Antibiotic class	Compliance (%)	OR	*p*-value
blaTEM	β-Lactams	88.9	9.78	0.001
blaZ	β-Lactams	100.0	98.53	<0.001
OXA	β-Lactams	83.3	5.00	0.056
StrA	Aminoglycosides	14.3	0.74	1.000
StrB	Aminoglycosides	37.5	3.22	0.149
aac(6)-aph2	Aminoglycosides	100.0	47.92	0.004
aadB	Aminoglycosides	100.0	74.68	<0.001
aph(3)	Aminoglycosides	25.0	1.71	0.536
aphA1	Aminoglycosides	42.9	4.09	0.105
dfrG	Sulfonamides	–	–	–
dfrK	Sulfonamides	–	–	–
ermC	Macrolides	100.0	33.86	0.003
ermB	Macrolides	75.0	10.36	0.052
mecA	β-lactams	-	-	-
qepA	Fluoroquinolones	100.0	9.00	0.117
qnrA	Fluoroquinolones	100.0	16.30	0.012
sul1	Sulfonamides	100.0	45.14	<0.001
sul3	Sulfonamides	100.0	45.14	<0.001
tetA	Tetracyclines	100.0	27.67	<0.001
tetB	Tetracyclines	90.0	12.21	0.006
tetK	Tetracyclines	100.0	37.22	<0.001
tetM	Tetracyclines	75.0	5.65	0.145

High concordance between the genotype and phenotype was revealed for β-lactams: blaTEM and blaZ showed a compliance of 88.9–100% (OR 9.78–98.53; *p* < 0.001). Significant overlap was also noted for the aminoglycoside-associated determinants aac(6)-aph2 and aadB (100% compliance; p < 0.01), as well as for the genes sul1, sul3, tetA, tetB and tetK, which confer resistance to sulfonamides and tetracyclines (90–100% compliance; *p* < 0.01). The qnrA and ermC genes were also significantly associated with resistance to fluoroquinolones and macrolides, respectively. Thus, most of the studied determinants have high diagnostic significance for predicting phenotypic resistance in bacterial isolates from dogs.

### 3.6 Identification of *E. coli* serogroups and *Staphylococcus aureus* enterotoxin genes (a to E)

Chromogenic media were used to isolate and identify *E. coli* STEC and O157, enabling clear differentiation between Shiga toxin-producing *E. coli* (STEC) and serotype O157: H7 verotoxin-producing *E. coli*.

Among the 65 *E. coli* isolates, Shiga toxin-producing strains (STECs) were identified in six cases (9.2%)—four strains from dogs with parvoviral enteritis and two from clinically healthy animals. In addition, *E. coli* O157: H7—a strain of high epidemiological significance—was detected in one dog with confirmed CPV-2 infection.

The enterotoxigenic properties of the *S. aureus* isolates were evaluated using an enzyme-linked immunosorbent assay (ELISA). Differences were observed in the ability to produce enterotoxins A, B, C, D, and E. Among the 53 *S. aureus* isolates, the ability to produce enterotoxins (SEA-SEE) was detected in two strains (3.8%), both of which were isolated from dogs with parvoviral enteritis, with one producing enterotoxin D and the other, enterotoxin E. The remaining isolates (*n* = 51; 96.2%) were nonenterotoxigenic.

## 4 Discussion

Despite a significant decrease in incidence due to the widespread introduction of vaccination, canine parvovirus infection (CPV-2) continues to be a serious global threat to domestic and wild carnivores. Most previous studies have focused mainly on characterizing CPV-2 and analysing its spread in different regions of the world, while data on concomitant bacterial infections, their role in exacerbating the clinical course, and antimicrobial resistance profiles remain limited (2).

This study revealed a high susceptibility of young unvaccinated dogs to canine parvovirus (CPV-2) infection; among 2,831 dogs under the age of 12 months, 7% tested positive. Death was reported in 38 of the 198 dogs (19.2%), and these data are consistent with the results of studies demonstrating that a lack of vaccination is among the key risk factors for CPV-2 infection in puppies (15). Notably, 80% of the infected animals were not vaccinated, and the remaining 20% developed clinical manifestations of the disease after a single vaccination in the series. Although bacterial coinfection was more common in nonfatal cases, there was no statistically significant association between the presence of bacterial isolates and mortality (OR = 1.31; *p* = 0.27). This result may be due to the provision of timely therapeutic support in a hospital setting. This distribution highlights the critical importance of fully implementing primary and booster vaccination regimens (40).

In this study, opportunistic pathogens were detected in significant proportions of dogs with confirmed CPV-2 infection and clinically healthy animals. The dominance of *S. aureus* (40.5%) is consistent with the literature regarding the high prevalence of this pathogen in dogs (41). The frequency of release of *E. coli* and *Klebsiella* spp. was comparable to the results of studies conducted in Europe and Asia (42–44). The differences between the regions are probably due to differences in animal husbandry and the methods of microbiological diagnostics. Thus, both CPV-2-positive and healthy dogs in northern Kazakhstan remain important reservoirs of opportunistic and potentially resistant microflora.

Comparison of the isolation frequency between the groups revealed that in dogs with CPV-2 (*n* = 198), gram-negative bacteria were isolated slightly more often than in clinically healthy animals (35.4% vs. 29.5%; *χ*^2^ = 1.32; *p* = 0.25; 95% CI: −4.2 − +15.0 P. P.). Thus, although higher absolute values of bacterial isolation frequency were observed in the CPV-2^+^ group, the difference was not statistically significant. From a biological perspective, this trend may be due to disruption of the intestinal barrier and an increased bacterial load in the presence of CPV-2 infection, which is consistent with the literature but requires confirmation in a larger sample (2, 45).

Of particular importance is the methodological approach that excludes the use of antibiotics before admission to the clinic. This approach allowed us to obtain an undisturbed picture of the natural intestinal microbiota and objectively evaluate the profiles of antimicrobial resistance in dogs infected with CPV-2. The exclusion of preexposure to antibiotics is critical, since even short-term therapy can significantly change the composition of the microflora and increase the proportion of resistant microbes (46, 47).

Owing to this strict treatment control, we obtained more accurate data on the true bacterial landscape in animals infected with CPV-2 and on the natural resistance level of circulating strains. Similar conclusions are presented in the work of Baker et al. (48), who reported that 55.6% of the studied dogs had received antibiotics in the 12 months before hospitalization, which significantly complicates the interpretation of the results of microbiological studies. Standardization of the conditions and exclusion of antibiotic therapy before the collection of material increase the accuracy of the analysis and allow more reliable data on natural antimicrobial resistance in veterinary populations to be obtained.

A high frequency of coinfections with opportunistic bacteria (*E. coli*, *Klebsiella* spp., and *S. aureus*) was detected in dogs infected with CPV-2B, consistent with current data on intestinal barrier disorders and bacterial translocation in parvovirus enteritis (2). This finding highlights the importance of CPV-2 as a factor in the formation of a reservoir of AMR-associated microorganisms.

*E. coli* coinfection was accompanied by the most severe gastrointestinal and metabolic disorders, consistent with the data on the role of gram-negative bacteria in the development of a systemic inflammatory response in the context of CPV-2 infection (49). Coinfection with *Klebsiella* spp. was associated with more pronounced signs of intoxication and multiple organ dysfunction, which was confirmed by international observations of its high virulence in the setting of immunodeficiency (50). Moreover, *S. aureus* manifested in most animals as a secondary inflammatory agent, prolonging recovery without severe systemic complications (51).

The results indicate that the severity of the course of parvovirus infection and the likelihood of bacterial coinfection may be related to the load of CPV-2 (2, 52). Although quantitative determination of the viral titre was not performed in this study, the observed clinical patterns are consistent with the literature, where increased viral replication is associated with pronounced destruction of the intestinal epithelium and profound immunodeficiency. This mechanism creates favourable conditions for the translocation of opportunistic microflora (in particular, *E. coli, Klebsiella* spp., and *Staphylococcus* spp.) and increases the likelihood of septic complications (2, 52, 53).

The lack of bacterial growth in some animals may reflect less pronounced mucosal destruction or an earlier stage of the disease before secondary bacterial translocation has occurred (2). This finding highlights the significant variability in the pathogenetic mechanisms of CPV-2 and highlights the role of the bacterial component in determining the severity of the disease. From a practical point of view, the revealed patterns emphasize the need for early detection of bacterial pathogens and the rational use of antimicrobial agents, considering local resistance profiles, especially in regions with a high prevalence of resistance genes (53). The integration of data on the viral load, severity of epithelial damage, and nature of bacterial translocation suggests that the combination of these factors determines the clinical severity of the disease and prognosis of the patient.

In general, our results, combined with data from the literature, suggest that the load of CPV-2 and the degree of bacterial coinfection are interdependent factors that reinforce each other in the pathogenesis of parvovirus enteritis. To confirm the identified trends, further studies are needed to quantify the viral titre and stratify animals according to the level of viremia and the severity of bacterial translocation.

Since the severity of clinical manifestations and the severity of CPV-2 infection are largely determined not only by the viral agent but also by the coinfecting bacteria, a detailed analysis of the resistance of isolated microorganisms to antimicrobial drugs was carried out. This analysis is particularly important given the increasing prevalence of antibiotic-resistant strains among pet pathogens, as noted in a number of foreign studies (54–56).

In a study of *E. coli* isolated from dogs with CPV-2, the strains demonstrated high rates of resistance to tetracyclines (58.4%), beta-lactams (34.5%) and fluoroquinolones (51.2%). These results are consistent with data on concomitant infections in companion animals in Europe, where the use of antibiotics is an important factor in the selection of resistant isolates (56). The lower rates in the group of clinically healthy animals confirm that CPV-2 infection increases breeding pressure and contributes to an increase in the proportion of resistant *E. coli* isolates. Similar trends in MDR have been described in dogs in South Korea (ampicillin AMR rate—38.3%; tetracycline -– 23.1%) (57) and in Spain, where approximately 50% of *E. coli* strains are characterized by multidrug resistance (58).

Compared with healthy animals, the group of dogs coinfected with CPV-2 and *Klebsiella* spp. was also characterized by significantly more pronounced resistance to the main classes of antibiotics. This finding is consistent with the results of European studies: in Germany, resistance in *K. pneumoniae* isolates has significantly increased among domestic animals in recent years (59). Additional confirmation of the high incidence of MDR among *Klebsiella* in dogs was obtained in Bulgaria (60). Thus, our data confirm the global trend according to which *Klebsiella* spp. form a population of aggressive, therapeutically complex isolates under conditions of viral immunodeficiency.

*S. aureus* isolates from dogs with CPV-2-induced enteritis also showed resistance to tetracyclines (48.3%), macrolides (27.6%) and *β*-lactams (~31%). These data are comparable with data from studies in Asia on staphylococcus resistance in animals (61) and from European studies, which emphasize the continued resistance of *Staphylococcus* spp. to the main classes of antibiotics used in veterinary medicine (60, 62).].

After phenotypic resistance was analysed, molecular markers were identified to identify the resistance mechanisms of the microorganisms and their differences between the groups. Molecular analysis confirmed a marked difference in antibiotic resistance profiles between bacteria isolated from dogs with CPV-2 and those isolated from clinically healthy animals. The genes associated with resistance to beta-lactams and aminoglycosides had the highest detection rates, consistent with current global trends in the spread of AMR among the microbiota of domestic animals (63, 64).

*E. coli* isolates from CPV-2-positive dogs were characterized by higher detection rates of plasmid beta-lactamases (blaTEM and OXA) as well as determinants of resistance to tetracyclines (tetB) and aminoglycosides (StrA/B) than those of isolates from clinically healthy animals. These differences are consistent with data from European studies, where the active circulation of these genes in the Enterobacteriaceae population in companion animals was noted (64, 65). This highlights the idea that a virus-induced immunosuppressive state can enhance the selection of multidrug-resistant genotypes.

The resistance profile of *Klebsiella* spp. was the most aggressive: a combination of ESBL- and OXA-mediated mechanisms, aminoglycoside genes (aphA1) and determinants of macrolide/quinolone resistance (qepA) were detected significantly more often in dogs with CPV-2 infection than in clinically healthy dogs. Comparisons with data from studies conducted in Asia confirmed the increasing importance of plasmid-mediated transmission of AMR genes in these strains (61). The high proportion of multidrug-resistant *Klebsiella* isolates increases the risk of severe viral and bacterial coinfections in the study region.

The blaZ gene was detected more frequently in *S. aureus* than in the other tested bacteria, whereas mecA was not detected in any sample, indicating that MRSA was not prevalent among dogs in northern Kazakhstan, consistent with reports from other countries (66, 67). Moreover, the more frequent detection of the tetK/M and erm genes in sick dogs was consistent with the global trend of an increasing number of resistant staphylococci under conditions of coinfection (62, 68).

The present study revealed that for most of the key antibiotic resistance genes, the degree of correspondence with the phenotypic resistance profiles was high. A particularly pronounced correlation was established for *β*-lactamases (blaTEM and blaZ), aminoglycoside-modifying enzymes (aac(6)-aph2 and aadB), sulfonamide-associated determinants (sul1 and sul3) and tetracycline resistance genes (tetA, tetB, and tetK), which highlights their clinical significance and the possibility of their use as molecular markers of phenotypic resistance.

The identification of the qnrA and ermC genes among dog isolates indicates the circulation of determinants of resistance to fluoroquinolones and macrolides, which pose an epidemiological risk within the One Health framework. However, the absence of isolates positive for mecA and for the trimethoprim resistance genes dfrG and dfrK limited the possibility of assessing their contribution.

Notably, the study did not include extended screening of ESBL genes (for example, blaCTX-M and blaSHV), which play a critical role in the spread of resistance among representatives of the Enterobacteriaceae family. This limitation may have led to underestimation of the true level of beta-lactamase resistance, and further extensive molecular studies are needed.

The obtained molecular data confirm that Central Asia constitutes a clinically significant reservoir of AMR-associated bacteria in dogs that requires systematic monitoring and that the resistance profile in the region is comparable to or even more pronounced than that in a number of European and Asian countries, which underscores the need to review approaches to empirical therapy in veterinary medicine.

From a practical point of view, the results emphasize the need for a more balanced approach to antibacterial therapy for parvovirus enteritis. The use of antibiotics is justified primarily when clinical and laboratory signs of bacterial complications (fever, severe leukopaenia or neutropaenia, signs of sepsis, or confirmed translocation of gram-negative bacteria) are present, as well as in patients in high-risk groups (puppies, animals with severe dehydration, animals with hypoproteinaemia). For other cases, it is advisable to shift the focus to intensive infusion and symptomatic and nutritional support, thus minimizing the use of systemic antimicrobials without clear indications.

When choosing an antibacterial therapy regimen for dogs with CPV-2 infection, it is advisable to rely on local data on the sensitivity of pathogens and avoid the routine use of third- and fourth-generation cephalosporins and fluoroquinolones as first-line drugs, especially in patients with mild and moderate disease. Preference should be given to beta-lactams with a narrower spectrum of action and subsequent de-escalation of therapy based on the results of bacteriological analysis. Patient management based on the principles of antimicrobial stewardship, regular updating of local antibiotic charts and documentation of cases of multidrug resistance should be considered key elements of clinical practice in a region where dogs are in close contact with humans and can act as an important link in the transmission of AMR within the One Health framework.

Thus, our study highlights the need for systemic epidemiological and molecular monitoring of CPV-2 and concomitant bacterial infections; the introduction of rational antibiotic therapy, considering local resistance profiles; and the development of antimicrobial resistance control programs among domestic animals. The data obtained have not only scientific value but also significant practical value for veterinary clinical practice and surveillance.

## 5 Conclusion

The results of this study confirm the relevance of parvovirus enteritis and the important role of coinfections with bacteria exhibiting a high level of antimicrobial resistance in puppies. This study constitutes the first collection of these data in Kazakhstan and complements the international surveillance of CPV-2 and AMR.

The key contribution of this research lies in its international significance. First, the data obtained from Central Asia help to bridge the existing geographical gap and increase the external validity of the global CPV-2 and AMP estimates. Second, the cross-border movement of animals and potential wildlife reservoirs make AMP profiles and circulating resistance genes a matter of global biosafety rather than a purely local problem. Third, the consistency of the results of our phenotype–genotype models with trends observed in Europe and Asia confirms that harmonized surveillance and the rational use of antibiotics should be based on standardized testing panels and uniform interpretation criteria.

Strengthening epizootic control and expanding vaccination coverage remain the main strategies for preventing CPV-2 infection. Our data emphasize the urgency of the problem of parvovirus enteritis in young dogs and the importance of a differentiated approach to therapy. Antibiotics should be prescribed strictly rationally—only for dogs with confirmed bacterial coinfection or neutropaenia, with mandatory consideration of the local antibiotic resistance profile. Minimizing the use of fluoroquinolones and fourth-generation cephalosporins, as well as implementing the principles of rational antibiotic therapy, are key measures to reduce mortality and limit the spread of multidrug-resistant strains in veterinary practice in a region where dogs are in close contact with humans and can act as an important link in the transmission of AMR within the One Health framework.

## Data Availability

The original contributions presented in the study are included in the article/supplementary material, further inquiries can be directed to the corresponding authors.
